# The predictive value of trunk/leg fat ratio for type 2 diabetes mellitus remission after bariatric surgery: A new observation and insight

**DOI:** 10.3389/fendo.2022.1068917

**Published:** 2022-11-08

**Authors:** Beibei Cui, Weizheng Li, Guohui Wang, Pengzhou Li, Liyong Zhu, Shaihong Zhu

**Affiliations:** Department of General Surgery, Third Xiangya Hospital, Central South University, Changsha, China

**Keywords:** trunk/leg fat ratio, body fat distribution, diabetes remission, type 2 diabetes mellitus, bariatric surgery, predictor

## Abstract

**Background:**

Emerging evidence supported the significant role of body composition and fat distribution in the etiology and pathogenesis of Type 2 diabetes mellitus (T2DM).

**Objective:**

To assess the predictive value of representative parameters of body composition and fat distribution for T2DM remission after bariatric surgery.

**Methods:**

A total of 72 patients with T2DM who underwent bariatric surgery in our center between September 2010 and December 2018 were included in this retrospective observational study. Diabetes remission was defined according to the American Diabetes Association criteria released in 2021. Body fat percentage, skeletal muscle index, Android/Gynoid ratio and trunk/leg fat ratio were derived from dual-energy X-ray absorptiometry and assessed.

**Results:**

A total of 40 patients (56%) achieved remission among 72 patients. Patients in the remission group had higher body fat percentage and lower trunk/leg fat ratio than those in the non-remission group. The area under the receiver operating characteristic curve (AUC) for predicting T2DM remission was higher for trunk/leg fat ratio (0.784), compared to BMI (AUC 0. 0.690) and body fat percentage (AUC 0.688). The prediction model (AUC 0.883) including age, duration of T2DM, and trunk/leg fat ratio performed better than the ABCD score (AUC 0.809) and the DiaRem score (AUC 0.792). A nonlinear relationship was observed between trunk/leg fat ratio and BMI.

**Conclusion:**

Trunk/leg fat ratio is a promising predictor for T2DM remission after bariatric surgery.

## Introduction

According to the International Diabetes Federation Atlas 10th edition, 537 million adults (20-79 years) are living with diabetes, which causes an estimated USD 966 billion in global health expenditure and 6.7 million deaths in 2021, and Type 2 diabetes mellitus (T2DM) accounts for over 90% of all diabetes worldwide (1). Accumulating evidence indicated that T2DM remission might sometimes be possible with the great improvement of therapies targeting metabolic control in T2DM (2). In a recent consensus statement, the American Diabetes Association (ADA) suggested that patients with T2DM should be considered in remission when sustaining a level of HbA_1c_ < 6.5% (48 mmol/mol) after cessation of glucose-lowering pharmacotherapy for at least 3 months (3).

Substantial evidence has proven that bariatric surgery is an effective treatment for T2DM, hence the ADA recommended bariatric surgery as the optimal treatment for patients with T2DM and severe obesity in 2017 (4, 5). Roux-en-Y gastric bypass (RYGB) and sleeve gastrectomy (SG) are the 2 most commonly performed bariatric procedures (6). A previous meta-analysis of randomized controlled trials indicated that about 60% of patients with T2DM achieved remission at 1 year after bariatric surgery and the available data seemed to support equal effects of both RYGB and SG in T2DM remission at 1 year after surgery, but the data beyond 3 years was insufficient (7). With the popularity of bariatric surgery in patients with T2DM, various predictors have been assessed to estimate the likelihood of T2DM remission after bariatric surgery (8–10). For example, higher levels of presurgical body mass index (BMI) and fasting C-peptide, a shorter duration of T2DM, younger, a lower level of HbA1c, no use of insulin, and no use of sulfonylureas or insulin-sensitizing agents other than metformin are predictors of an increased likelihood of T2DM remission after bariatric surgery.

Emerging evidence supported the significant role of body composition and fat distribution in the etiology and pathogenesis of T2DM (11, 12). It has been widely established that the accumulation of visceral adipose tissue is a powerful predictor to estimate the risk of T2DM (13, 14). Further studies confirmed that trunk fat and leg fat had independent and opposite associations with glucose metabolism and T2DM (15, 16), while fat-free mass and muscle mass served as predictors for T2DM remission after bariatric surgery (17). This study aimed to assess the predictive value of representative parameters of body composition and fat distribution for T2DM remission after bariatric surgery.

## Methods

### Study design and patients

This is a retrospective observational study to examine the predictive value of representative parameters of body composition and fat distribution for T2DM remission after bariatric surgery. The institutional review board of the hospital approved the study and each patient provided written informed consent. The inclusion criteria were (1) age 18-65 years, (2) T2DM diagnosed with the latest ADA diagnostic criteria (18), (3) BMI > 25 kg/m^2^, and (4) T2DM duration < 15 years. The exclusion criteria were (1) serious T2DM complications or organic diseases, (2) alcohol addiction, or (3) unstable mental disorders. We searched the electronic medical records system and identified 89 consecutive individuals with T2DM who underwent RYGB or SG between September 2010 and December 2018 and simultaneously received presurgical DXA scans. All patients underwent bariatric surgery from the same surgical team and were under the same medical care, including dietary and exercise counseling. According to the latest ADA consensus statement released in 2021, patients with T2DM should be considered in remission when sustaining a level of HbA_1c_ < 6.5% (48 mmol/mol) or a level of fasting plasma glucose < 126 mg/dL (7.0 mmol/L) after cessation of glucose-lowering pharmacotherapy for at least 3 months, while an interval of at least 3 months is required for the time of initiation after bariatric surgery (3), thus 17 patients with a poor follow-up (less than 6 months) were excluded because of failing to confirm T2DM remission. Finally, 72 patients were included in the study.

### Variables and data collection

The data on age, sex, BMI, waist-to-hip ratio, representative parameters of body composition (body fat percentage [BF%] and skeletal muscle index [SMI]) and fat distribution (Android/Gynoid ratio and trunk/leg fat ratio), blood pressure, indexes of glucose homeostasis (fasting, 30-min and 120-min plasma glucose, insulin, C-peptide, and HbA1c), indexes of lipid profile (HDL cholesterol, LDL cholesterol, triglyceride, and total cholesterol), liver function (Alanine transaminase, Aspartate transaminase, total bilirubin, direct bilirubin, total protein, Albumin protein, Globulin protein, total bile acid), renal function (blood uric nitrogen and blood creatinine), medical history (duration of T2DM, hypoglycemic agent history, and insulin use) were identified and collected. Android/Gynoid ratio was Android fat divided by Gynoid fat. Trunk/leg fat ratio was trunk fat divided by leg fat. BF% was calculated according to the following formula: BF% = total fat mass/total body mass × 100%. SMI was defined as the sum of the muscle mass in both arms and legs divided by height squared. Body composition and fat distribution were assessed by dual-energy X-ray absorptiometry (LUNAR DPX NT+ 74029, GE Medical System, USA).

### Statistical analysis

Data were summarized as mean ± SD for quantitative variables, or count and percentage for qualitative variables. The unpaired t test or the Mann-Whitney U test were used to investigate differences between the remission group and the non-remission group for quantitative variables, as appropriate, while the Fisher test was used for qualitative variables. The paired t test or the Wilcoxon signed-rank test were used to compare baseline and follow-up data, as appropriate. Briefly, we followed a four-step process to identify potential predictors and the optimal predictive model: (1) variable reduction: univariate logistic regression analysis was performed to reduce the redundant variables and filter out those with weak predictive power; (2) variable transformation: the receiver operating characteristic (ROC) curve was used to determine the optimal cut-off of quantitative variables and transform quantitative variables into binary variables; (3) model development: multiple logistic regression analysis was performed to judge the independent association of each “prime candidate” and T2DM remission, and K-fold cross validation (CV), Akaike information criterion (AIC), and Bayesian information criterion (BIC) were further used to determine the optimal model to predict T2DM remission (19, 20); (4) model assessment: the area under the receiver operating characteristic curve (AUC) was used to compared the performances of different models. The break point analysis was used to explore the nonlinear relationship between quantitative variables. In addition, we used a Cox proportional hazards model to estimate the hazard ratios (HRs) of the optimal predictors (from the optimal model) for diabetes remission. We also created Kaplan-Meier curves of time to diabetes remission. Patients who failed to achieve remission were censored. All analyses were performed using R software version 4.1.2 (http://www.r-project.org/) and two-tailed *P* values of < 0.05 were considered to indicate statistical significance.

## Results

### Patient demographics and clinical characteristics

A total of 40 patients (56%) achieved remission among 72 patients, of which 43 patients (60%) underwent RYGB. The comparisons of demographics and clinical characteristics between the remission group and the non-remission group were listed in **Table 1**. The median follow-up time was 13 (6-31) months. The comparisons of demographics and clinical characteristics between baseline and follow-up points were listed in **Table 2**.

**Table 1:** Patient demographics and clinical characteristics at baseline.

Variables	Remission (n = 40)	Non-remission (n = 32)	*P*
Age, years	37.5 ± 11.2	47.2 ± 7.9	**<0.001**
Women, n (%)	11 (27.5)	8 (25.0)	0.811
Anthropometric measurements			
BMI, kg/m^2^	33.2 ± 4.6	30.7 ± 5.3	**0.006**
Body fat percentage, %	36.2 ± 6.4	32.1 ± 6.6	**0.011**
Skeletal muscle index, kg/m^2^	8.5 ± 1.0	8.3 ± 1.4	0.538
Waist-to-hip ratio	0.99 ± 0.06	0.97 ± 0.06	0.159
Android/Gynoid ratio	0.82 ± 0.15	0.88 ± 0.20	0.149
Trunk/leg fat ratio	2.31 ± 0.54	3.05 ± 0.81	**<0.001**
Blood pressure			
Diastolic blood pressure, mmHg	90.4 ± 14.3	86.4 ± 11.0	0.240
Systolic blood pressure, mmHg	137.8 ± 19.5	134.9 ± 15.5	0.555
Glucose homeostasis			
Fasting plasma glucose, mmol/L	8.1 ± 3.1	9.3 ± 3.5	0.147
30-min plasma glucose, mmol/L	13.4 ± 3.9	13.0 ± 4.1	0.702
120-min plasma glucose, mmol/L	15.3 ± 5.5	17.5 ± 4.5	0.073
Fasting insulin, uU/mL	18.3 ± 16.6	19.6 ± 21.2	0.669
30-min insulin, uU/mL	41.8 ± 35.1	30.1 ± 26.5	0.144
120-min insulin, uU/mL	72.6 ± 53.3	40.2 ± 29.9	**0.003**
Fasting C-peptide, ng/mL	3.3 ± 2.0	2.1 ± 1.2	**0.002**
30-min C-peptide, ng/mL	4.9 ± 3.1	3.2 ± 2.2	**0.009**
120-min C-peptide, ng/mL	9.5 ± 7.5	4.9 ± 2.6	**<0.001**
HbA1c, %	8.3 ± 1.9	8.6 ± 1.7	0.437
Lipid profile			
HDL cholesterol, mmol/L	1.1± 0.2	1.1 ± 0.2	0.836
LDL cholesterol, mmol/L	2.6 ± 1.0	2.5 ± 0.6	0.594
Triglycerides, mmol/L	2.5 ± 1.8	3.1 ± 3.0	0.527
Total cholesterol, mmol/L	4.9 ± 1.3	4.9 ± 1.1	0.910
Liver function			
Alanine transaminase, U/L	59.4 ± 52.7	39.1 ± 32.0	0.052
Aspartate transaminase, U/L	35.8 ± 24.6	28.6 ± 19.1	0.134
Total bilirubin, umol/L	13.5 ± 7.8	12.8 ± 5.6	0.874
Direct bilirubin, umol/L	4.5 ± 3.2	3.3 ± 1.5	0.223
Total protein, g/L	70.1 ± 5.9	71.0 ± 7.9	0.596
Albumin protein, g/L	43.8 ± 4.7	42.8 ± 4.6	0.340
Globulin protein, g/L	26.2 ± 3.8	28.2 ± 5.0	0.065
Total bile acid, umol/L	5.2 ± 3.6	7.5 ± 9.0	0.245
Renal function			
Blood uric nitrogen, mmol/L	4.7 ± 1.7	5.2 ± 1.6	0.217
Blood creatinine, umol/L	67.0 ± 18.3	72.5 ± 28.9	0.511
Medicine			
Sulfonylureas or insulin sensitizing agents other than metformin (yes), n (%)	15 (37.5)	18 (56.3)	0.113
Insulin use (yes), n (%)	12 (30.0)	24 (75.0)	**<0.001**
Duration of Type 2 diabetes mellitus, years	4.1 ± 4.4	6.9 ± 3.9	**<0.001**
Surgery Type			
Sleeve gastrectomy	25 (62.5)	4 (12.5)	**<0.001**
Roux-en-Y gastric bypass	15 (37.5)	28 (87.5)	

Quantitative variables are presented as mean ± SD. Qualitative variables are presented as n (%). P values in boldface are statistically significant. BMI, Body mass index; HDL, high-density lipoprotein; LDL, low-density lipoprotein.

**Table 2:** Variables in the preoperative and postoperative periods.

	Baseline	<2 years	≥2 years
Anthropometric measurements			
BMI, kg/m2	32.1 ± 5.0	26.4 ± 4.0*	26.4 ± 3.6*
Waist-to-hip ratio	0.98 ± 0.06	0.94 ± 0.06*	0.96 ± 0.05
Blood pressure			
Diastolic blood pressure, mmHg	88.6 ± 13.0	83.2 ± 12.2*	86.2 ± 12.7
Systolic blood pressure, mmHg	136.5 ± 17.8	130.2 ± 19.6*	130.2 ± 16.2
Glucose homeostasis			
Fasting plasma glucose, mmol/L	8.6 ± 3.3	6.0 ± 1.8*	6.6 ± 2.5*
30-min plasma glucose, mmol/L	13.2 ± 4.0	12.1 ± 3.1*	13.4 ± 4.0
120-min plasma glucose, mmol/L	16.3 ± 5.2	9.3 ± 4.8*	10.6 ± 4.8*
Fasting insulin, uU/mL	18.9 ± 18.8	9.6 ± 7.9*	9.4 ± 7.6*
30-min insulin, uU/mL	36.1 ± 31.6	86.1 ± 85.2*	72.6 ± 84.7*
120-min insulin, uU/mL	57.5 ± 46.6	39.0 ± 38.8*	31.6 ± 53.6
Fasting C-peptide, ng/mL	2.8 ± 1.8	2.4 ± 2.3*	3.1 ± 3.7
30-min C-peptide, ng/mL	4.1 ± 2.8	7.5 ± 5.3*	7.7 ± 6.2*
120-min C-peptide, ng/mL	7.4 ± 6.2	8.5 ± 8.3	8.4 ± 12.0
HbA1c, %	8.4 ± 1.8	6.5 ± 1.4*	7.4 ± 2.1
Lipid profile			
HDL cholesterol, mmol/L	1.1 ± 0.2	1.2 ± 0.3*	1.2 ± 0.3
LDL cholesterol, mmol/L	2.6 ± 0.8	2.4 ± 0.7	2.6 ± 0.8
Triglycerides, mmol/L	2.8 ± 2.4	1.3 ± 1.0*	1.5 ± 1.1*
Total cholesterol, mmol/L	4.9 ± 1.2	4.5 ± 0.9*	4.5 ± 1.0
Liver function			
Alanine transaminase, U/L	50.4 ± 45.6	23.5 ± 15.0*	25.8 ± 17.0*
Aspartate transaminase, U/L	32.6 ± 22.5	24.4 ± 32.5*	22.4 ± 9.4*
Total bilirubin, umol/L	13.2 ± 6.9	12.1 ± 5.2	12.2 ± 4.6
Direct bilirubin, umol/L	4.0 ± 2.6	3.6 ± 2.2	3.8 ± 1.6
Total protein, g/L	70.5 ± 6.8	67.5 ± 6.5*	68.1 ± 6.9
Albumin protein, g/L	43.4 ± 4.6	42.9 ± 4.2	43.4 ± 4.9
Globulin protein, g/L	27.1 ± 4.5	24.6 ± 4.6*	25.2 ± 3.1*
Total bile acid, umol/L	6.2 ± 6.7	8.7 ± 18.0	14.3 ± 36.9
Renal function			
Blood uric nitrogen, mmol/L	4.9 ± 1.6	4.9 ± 1.3	5.3 ± 2.0
Blood creatinine, umol/L	69.5 ± 23.6	67.3 ± 19.9	75.4 ± 37.8

Quantitative variables are presented as mean ± SD. BMI, Body mass index; HDL, high-density lipoprotein; LDL, low-density lipoprotein. *P < 0.05 compared with baseline.

### Variable reduction and transformation

**Figure 1:**
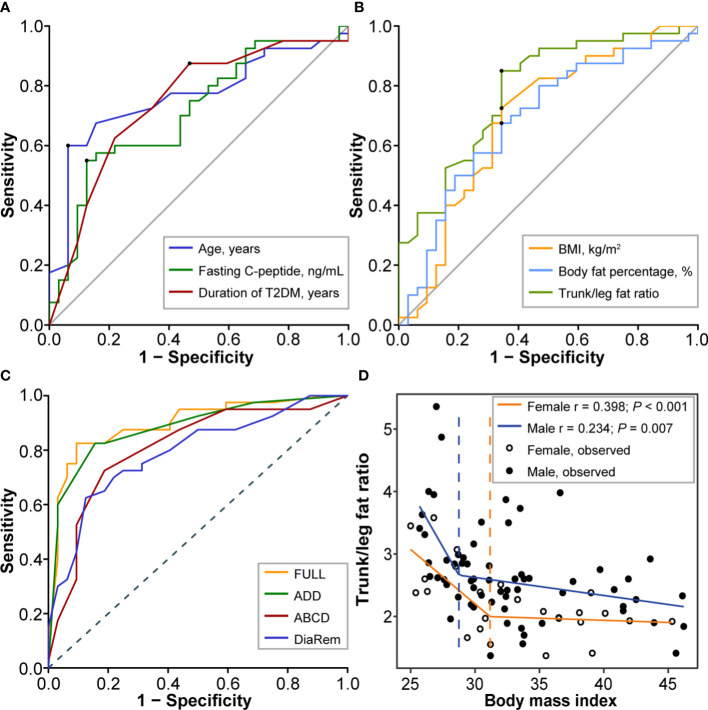
Screening and assessment of predictors. **(A)** The ROC curves for age (cutoff 39.0 years, sensitivity 0.600, specificity 0.938, the Youden index 0.538, AUC 0.762), fasting C-peptide (cutoff 3.1 ng/mL, sensitivity 0.550, specificity 0.875, the Youden index 0.425, AUC 0.711), and duration of T2DM (cutoff 6.5 years, sensitivity 0.875, specificity 0.0.531, the Youden index 0.406, AUC 0.744). **(B)** The ROC curves for BMI (cutoff 30.5 kg/m^2^, sensitivity 0.725, specificity 0.656, the Youden index 0.381, AUC 0.690), body fat percentage (cutoff 33.5%, sensitivity 0.675, specificity 0.656, the Youden index 0.331, AUC 0.688), and trunk/leg fat ratio (cutoff 2.63, sensitivity 0.850, specificity 0.656, the Youden index 0.516, AUC 0.784). **(C)** The ROC curves for the full model (AUC 0.896, 95%CI 0.821-0.972), the ADD tool (AUC 0.883, 95%CI 0.806-0.961), the ABCD score (AUC 0.809, 95%CI 0.705-0.913), and the DiaRem score (AUC 0.792, 95%CI 0.688-0.896) in predicting T2DM remission after bariatric surgery. **(D)** The nonlinear relationship between trunk/leg fat ratio and BMI. The break points showing a sharp change in slope are indicated by a dashed line with the corresponding color. Break points of BMI were respectively 28.75 kg/m^2^ (males) and 31.17 kg/m^2^ (females) for trunk/leg fat ratio. ROC, Receiver operating characteristic; AUC, Area under the curve; BMI, Body mass index; CI, Confidence interval; T2DM, Type 2 diabetes mellitus.

For variable reduction, univariate logistic regression analysis identified 8 variables (**Table 3**), of which 6 quantitative variables were then transformed into binary variables for subsequent multiple logistic regression analysis. For variable transformation, the optimal cutoff value of age was 39.0 years (sensitivity 0.600, specificity 0.938, the Youden index 0.538, AUC 0.762) and we selected cut-off as 40 years. The optimal cutoff value of fasting C-peptide was 3.1 ng/mL (sensitivity 0.550, specificity 0.875, the Youden index 0.425, AUC 0.711) and we selected cut-off as 3 ng/mL. The optimal cutoff value of duration of T2DM was 6.5 years (sensitivity 0.875, specificity 0.0.531, the Youden index 0.406, AUC 0.744) and we selected cut-off as 7 years (**Figure 1A**). The optimal cutoff value of BMI was 30.5 kg/m^2^ (sensitivity 0.725, specificity 0.656, the Youden index 0.381, AUC 0.690) and we selected cut-off as 30 kg/m2. The optimal cutoff value of BF% was 33.5% (sensitivity 0.675, specificity 0.656, the Youden index 0.331, AUC 0.688) and we selected cut-off as 33.5%. The optimal cutoff value of trunk/leg fat ratio was 2.63 (sensitivity 0.850, specificity 0.656, the Youden index 0.516, AUC 0.784) and we selected cut-off as 2.63 (**Figure 1B**).

**Table 3:** Logistic regression analyses for predictor screening and model building.

Variables	OR (95%CI)	*P*
Univariate logistic regression		
Age, years	0.90 (0.85-0.96)	**0.001**
Sex, men vs women	0.88 (0.30-2.53)	0.811
BMI, kg/m2	1.11 (1.00-1.24)	**0.049**
Body fat percentage, %	1.10 (1.02-1.19)	**0.014**
Skeletal muscle index, kg/m2	1.14 (0.77-1.69)	0.519
Waist-to-hip ratio	2533.35 (0.34-18711329.3)	0.085
Android/Gynoid ratio	0.12 (0.01-2.00)	0.140
Trunk/leg fat ratio	0.16 (0.06-0.43)	**<0.001**
Fasting C-peptide, ng/mL	1.78 (1.19-2.64)	**0.005**
HbA1c, %	0.92 (0.70-1.20)	0.543
Sulfonylureas or insulin sensitizing agents other than metformin, yes vs no	0.47 (0.18-1.20)	0.115
Insulin use, yes vs no	0.14 (0.05-0.41)	**<0.001**
Duration of Type 2 diabetes mellitus, years	0.84 (0.74-0.96)	**0.011**
Surgery type, RYGB vs SG	0.09 (0.03-0.29)	**<0.001**
Multiple logistic regression (full model)		
Age, years, ≥40 vs <40	0.08 (0.01-0.56)	**0.010**
BMI, kg/m2, ≥30 vs <30	0.89 (0.18-4.50)	0.885
Body fat percentage, %, ≥33.5 vs <33.5	0.91 (0.20-4.23)	0.908
Trunk/leg fat ratio, ≥2.63 vs <2.63	0.29 (0.05-1.69)	0.169
Fasting C-peptide, ng/mL, ≥3 vs <3	0.56 (0.09-3.75)	0.554
Insulin use, yes vs no	0.51 (0.10-2.71)	0.430
Duration of Type 2 diabetes mellitus, years, ≥7 vs <7	0.21 (0.04-1.12)	0.069
Surgery type, RYGB vs SG	0.36 (0.07-2.01)	0.246
Best model selected by CV, BIC, and AIC (ADD tool)		
Age, years, ≥40 vs <40	0.09 (0.02-0.53)	**0.007**
Duration of Type 2 diabetes mellitus, years, ≥7 vs <7	0.17 (0.04-0.70)	**0.014**
Trunk/leg fat ratio, ≥2.63 vs <2.63	0.18 (0.05-0.67)	**0.010**

P values in boldface are statistically significant. BMI, Body mass index; RYGB, Roux-en-Y gastric bypass; SG, Sleeve gastrectomy; CV, Cross validation; AIC, Akaike information criterion; BIC, Bayesian information criterion.

### Model development and assessment

For model development, we firstly including all 8 identified variables in a multiple logistic regression analysis (the full model). For the full model, the AIC score was 75.241 and the C-statistic was 0.896 (95%CI 0.821-0.972), while the odds of diabetes remission were lower in patients with an age ≥40 years (odds ratio [OR] 0.08, 95%CI [confidence interval] 0.01-0.56, *P* = 0.010, **Table 2**). According to CV, BIC, and AIC, the optimal subset including age, duration of T2DM, and trunk/leg fat ratio was then determined to develop the ADD tool, where A stood for age, the first D for duration of T2DM, and the second D for distribution of body fat, namely trunk/leg fat ratio (**Table 2**). For the ADD tool, the AIC score was 67.189 (better than the full model 75.241) and the C-statistic was 0.883 (95%CI 0.806-0.961). For model assessment, we compared the performances of the ADD tool with the full model, the ABCD score, and the DiaRem score. Based on the ROC curves, the full model had an AUC of 0.896 (95%CI 0.821-0.972), the ADD tool 0.883 (95%CI 0.806-0.961), the ABCD score 0.809 (95%CI 0.705-0.913), and the DiaRem score 0.792 (95%CI 0.688-0.896, **Figure 1C**). There was no significant difference between the ADD tool and the full model (*P* = 0.856), but the ADD tool performed better than the ABCD score (*P* < 0.001) and the DiaRem score (*P* < 0.001).

### The nonlinear relationship between trunk/leg fat ratio and BMI

Based on the whole data of 89 consecutive individuals, the trunk/leg fat ratio showed a nonlinear relationship with BMI (*P* = 0.007 for males and < 0.001 for females), regardless of sex, when BMI exceeded a certain threshold, the decrease in trunk/leg fat ratio slowed. Break points of BMI were respectively 28.75 (males) and 31.17 kg/m^2^ (females) for trunk/leg fat ratio (**Figure 1D**).

### Cox proportional hazards model and Kaplan-Meier curves

**Figure 2:**
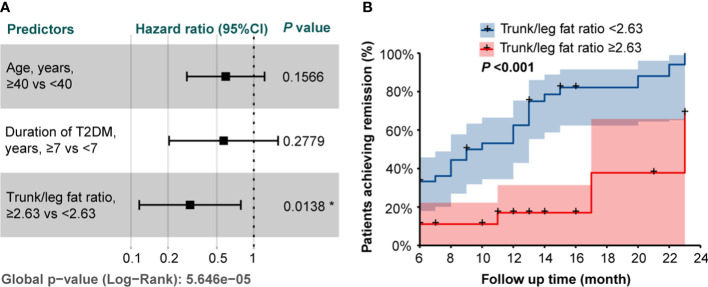
**(A)** The forest plot of the hazard ratios from the Cox proportional hazards model including age, duration of T2DM, and trunk/leg fat ratio. * indicated statistical significance (P < 0.05). **(B)** Kaplan-Meier curves stratified by trunk/leg fat ratio with a log-rank test.

Based on the Cox proportional hazards model including age, duration of T2DM, and trunk/leg fat ratio, patients with a trunk/leg fat ratio ≥2.63 were less likely to achieve remission (HR 0.302, 95%CI 0.117-0.783; *P* = 0.0138, **Figure 2A**). The Kaplan-Meier curves with a log-rank test also supported patients with a trunk/leg fat ratio ≥2.63 were less likely to achieve remission (*P* < 0.001, **Figure 2B**).

## Discussion

In the present study, we assessed the predictive value of representative parameters of body composition and fat distribution for T2DM remission after bariatric surgery, and finally confirmed trunk/leg fat ratio as a promising predictor for T2DM remission after bariatric surgery. We further developed a prediction model, namely the ADD tool, which used age, duration of T2DM, and trunk/leg fat ratio to estimate the probabilities of T2DM remission after bariatric surgery. We compared predictive power of the ADD tool with the ABCD score and the DiaRem score (8, 9). We believe the ADD tool is scientific and efficient because it takes into account the general reserve of body function (age), the severity of presurgical T2DM (duration of T2DM), and the body shape-related risk of T2DM (trunk/leg fat ratio).

In agreement with previous models, the ADD tool also included patient age as a significant parameter (8, 9). As shown in both full model and ADD tool, patient age was the most powerful predictor. It was different from both the ABCD score and the DiaRem score, in which patient age was a relatively weak predictor. A possible explanation for this divergence is that T2DM displays a trend towards younger age groups (21, 22). In previous models, an age of younger than 40 years may correspond to shorter disease duration and earlier disease status and the age of 40 years may be the cut-off to distinguish between individuals who are easy to cure and those who are generally curable, whereas, with the increasing prevalence of T2DM among younger age groups, the age of 40 years may turn into the cut-off to distinguish between individuals who are generally curable and those who are hard to cure. In addition, both overweight and obesity, the significant risk factors of T2DM, also display a trend towards younger age groups, this increases the predictive weight of age in T2DM remission after bariatric surgery (23, 24).

In previous models, parallel surrogate variables indicating the severity of presurgical T2DM, such as duration of T2DM, the C-peptide level, requirement for multiple hypoglycemic agents or insulin use, have been proven as efficient predictors of T2DM remission after bariatric surgery (8, 9). In our study, duration of T2DM was finally identified in the optimal model, although the C-peptide level and insulin use were also significantly different between the remission group and the non-remission group according to univariate logistic regression analysis. As we all know, T2DM is characterized by a relative insulin deficiency and insulin resistance, while the C-peptide level is a good indicator of insulin secretion but is unable to reflect the degree of insulin resistance precisely. Insulin use is sometimes affected by prescription preferences of patients and clinicians. In this perspective, duration of T2DM should be a more objective and powerful predictor to reflect the severity of presurgical T2DM, to some degree, it indicates the integrated effects of insulin deficiency and insulin resistance. In accordance with our study, the ABCD score also highlighted the predictive power of duration of T2DM (8).

In contrast to previous models, the ADD tool takes into account the predictive power of body fat distribution in T2DM remission for the first time. Despite the BMI, a relatively simple surrogate for obesity, remaining the most important criterion in selecting candidates with T2DM for bariatric surgery, the predictive power of BMI has long been controversial. A previous meta-analysis including 4944 surgical patients with T2DM concluded that bariatric surgery resulted in similar remission rates in patients with a BMI ≥35 kg/m^2^ or a BMI <35 kg/m^2^ (25). In the 2nd Diabetes Surgery Summit, a multidisciplinary group including 48 international clinicians and scholars reached a unanimous agreement to develop and evaluate more appropriate criteria than BMI alone in selecting candidates with T2DM for bariatric surgery (26). Our study also indicated that presurgical BMI was not a strong enough predictor to be included in the optimal model. The nonlinear relationship between trunk/leg fat ratio and BMI may partially contribute to BMI limitations in predicting T2DM remission after bariatric surgery. Emerging evidence supported that body fat distribution, independent of overall fat mass, was strongly associated with the risk of T2DM (11, 27, 28). In some cases, overall fat mass returns an ambiguous prediction in the risk of T2DM. For example, some lean people unexpectedly share a similar risk of T2DM with those who suffer from overweight or obesity, while these two phenotypes share centripetal characteristics of fat distribution (29). Herein, we introduced trunk/leg fat ratio to gauge body fat distribution, taking into account both trunk fat (the main source of visceral adipose tissue) and leg fat (the main source of lower-body subcutaneous adipose tissue). In our prediction tool, patients with a trunk/leg fat ratio < 2.63 have approximately 6 times greater odds of achieving T2DM remission after bariatric surgery than those with a trunk/leg fat ratio ≥ 2.63. We believe this ratio can provide more accurate risk-benefit information than BMI.

Our study had several strengths. First, we confirmed the predictive value of trunk/leg fat ratio for T2DM remission after bariatric surgery based on the up-to-date ADA consensus statement on T2DM remission. Second, for the first time, body fat distribution was taken into consideration to predict the performance of bariatric surgery in T2DM treatment and the ADD tool including trunk/leg fat ratio was established. Third, a nonlinear relationship was observed between trunk/leg fat ratio and BMI, which may partially explain BMI limitations in predicting T2DM remission after bariatric surgery.

This study also had several limitations. First, the relatively small sample size might preclude us from adequately detecting the predictive power of some variables. However, the predictive value of trunk/leg fat ratio was confirmed from multiple perspectives: (1) trunk/leg fat ratio was significantly smaller in the remission group than in the non-remission group; (2) both univariate logistic regression analysis and ROC curve suggested a strong correlation between trunk/leg fat ratio and T2DM remission; (3) the ADD tool including trunk/leg fat ratio performed better than previous models; (4) the nonlinear relationship between trunk/leg fat ratio and BMI may partially explain BMI limitations in predicting T2DM remission after bariatric surgery; (5) both the Cox proportional hazards model and Kaplan-Meier curves supported trunk/leg fat ratio as a powerful predictor for T2DM remission. Second, trunk/leg fat ratio was derived from DXA which was a relatively expensive method. Confirming the consistency of DXA and bioelectrical impedance analysis or finding other alternative parameters of trunk/leg fat ratio may be the arenas for future research. Third, although the ADD tool performed well in the local data, its general performance needs to be further evaluated in additional data from different regions and ethnic groups.

## Conclusion

In conclusion, trunk/leg fat ratio is a promising predictor for T2DM remission after bariatric surgery. Trunk/leg fat ratio will enable clinicians and patients to evaluate the merits of bariatric surgery as an approach to achieve T2DM remission and determine if additional measures are necessary to enhance the remission odds.

## Data availability statement

The raw data supporting the conclusions of this article will be made available by the authors, without undue reservation.

## Ethics statement

The studies involving human participants were reviewed and approved by the Ethics Committee of the Third Xiangya hospital. The patients/participants provided their written informed consent to participate in this study.

## Author contributions

BC and WL performed all of the data analyses and wrote the manuscript; GW and PL obtained the data, interpreted the data and revised the manuscript critically. LZ and SZ designed and supervised the study, reviewed the data analyses, and edited the manuscript. All authors contributed to the discussion and approved the final manuscript. LZ and SZ are the guarantors of this work and, as such, had full access to all the data in the study and takes responsibility for the integrity of the data and the accuracy of the data analysis. All authors contributed to the article and approved the submitted version.

## Funding

This research was funded by the Wisdom Accumulation and Talent Cultivation Project of the Third Xiangya Hospital of Central South University, grant number YX202102.

## Conflict of interest

The authors declare that the research was conducted in the absence of any commercial or financial relationships that could be construed as a potential conflict of interest.

## Publisher’s note

All claims expressed in this article are solely those of the authors and do not necessarily represent those of their affiliated organizations, or those of the publisher, the editors and the reviewers. Any product that may be evaluated in this article, or claim that may be made by its manufacturer, is not guaranteed or endorsed by the publisher.

## References

[1] Sun H Saeedi P Karuranga S Pinkepank M Ogurtsova K Duncan BB. IDF diabetes atlas: Global, regional and country-level diabetes prevalence estimates for 2021 and projections for 2045. Diabetes Res Clin Pract (2022) 183:109119. doi: 10.1016/j.diabres.2021.109119 34879977 PMC11057359

[2] Buse JB Caprio S Cefalu WT Ceriello A Del Prato S Inzucchi SE. How do we define cure of diabetes? Diabetes Care (2009) 32(11):2133–5. doi: 10.2337/dc09-9036 PMC2768219 19875608

[3] Riddle MC Cefalu WT Evans PH Gerstein HC Nauck MA Oh WK. Consensus report: Definition and interpretation of remission in type 2 diabetes. Diabetes Care (2021) 44(10):2438–44. doi: 10.2337/dci21-0034 PMC8929179 34462270

[4] Schauer PR Bhatt DL Kirwan JP Wolski K Brethauer SA Navaneethan SD. Bariatric surgery versus intensive medical therapy for diabetes–3-Year outcomes. N Engl J Med (2014) 370(21):2002–13. doi: 10.1056/NEJMoa1401329 PMC5451259 24679060

[5] American Diabetes Association. 7. obesity management for the treatment of type 2 diabetes. Diabetes Care (2017) 40(Suppl 1):S57–s63. doi: 10.2337/dc17-S010 27979894

[6] English WJ DeMaria EJ Brethauer SA Mattar SG Rosenthal RJ Morton JM. American Society for metabolic and bariatric surgery estimation of metabolic and bariatric procedures performed in the united states in 2016. Surg Obes Relat Dis (2018) 14(3):259–63. doi: 10.1016/j.soard.2017.12.013 29370995

[7] Lee Y Doumouras AG Yu J Aditya I Gmora S Anvari M. Laparoscopic sleeve gastrectomy versus laparoscopic roux-En-Y gastric bypass: A systematic review and meta-analysis of weight loss, comorbidities, and biochemical outcomes from randomized controlled trials. Ann Surg (2021) 273(1):66–74. doi: 10.1097/sla.0000000000003671 31693504

[8] Lee WJ Hur KY Lakadawala M Kasama K Wong SK Chen SC. Predicting success of metabolic surgery: Age, body mass index, c-peptide, and duration score. Surg Obes Relat Dis (2013) 9(3):379–84. doi: 10.1016/j.soard.2012.07.015 22963817

[9] Still CD Wood GC Benotti P Petrick AT Gabrielsen J Strodel WE. Preoperative prediction of type 2 diabetes remission after roux-En-Y gastric bypass surgery: A retrospective cohort study. Lancet Diabetes Endocrinol (2014) 2(1):38–45. doi: 10.1016/s2213-8587(13)70070-6 24579062 PMC3932625

[10] Singh P Adderley NJ Hazlehurst J Price M Tahrani AA Nirantharakumar K. Prognostic models for predicting remission of diabetes following bariatric surgery: A systematic review and meta-analysis. Diabetes Care (2021) 44(11):2626–41. doi: 10.2337/dc21-0166 34670787

[11] Stefan N. Causes, consequences, and treatment of metabolically unhealthy fat distribution. Lancet Diabetes Endocrinol (2020) 8(7):616–27. doi: 10.1016/s2213-8587(20)30110-8 32559477

[12] Wagner R Heni M Tabák AG Machann J Schick F Randrianarisoa E. Pathophysiology-based subphenotyping of individuals at elevated risk for type 2 diabetes. Nat Med (2021) 27(1):49–57. doi: 10.1038/s41591-020-1116-9 33398163

[13] Neeland IJ Turer AT Ayers CR Powell-Wiley TM Vega GL Farzaneh-Far R. Dysfunctional adiposity and the risk of prediabetes and type 2 diabetes in obese adults. Jama (2012) 308(11):1150–9. doi: 10.1001/2012.jama.11132 PMC3556508 22990274

[14] Levelt E Pavlides M Banerjee R Mahmod M Kelly C Sellwood J. Ectopic and visceral fat deposition in lean and obese patients with type 2 diabetes. J Am Coll Cardiol (2016) 68(1):53–63. doi: 10.1016/j.jacc.2016.03.597 27364051 PMC4925621

[15] Snijder MB Dekker JM Visser M Bouter LM Stehouwer CD Yudkin JS. Trunk fat and leg fat have independent and opposite associations with fasting and postload glucose levels: The hoorn study. Diabetes Care (2004) 27(2):372–7. doi: 10.2337/diacare.27.2.372 14747216

[16] Vasan SK Osmond C Canoy D Christodoulides C Neville MJ Di Gravio C. Comparison of regional fat measurements by dual-energy X-ray absorptiometry and conventional anthropometry and their association with markers of diabetes and cardiovascular disease risk. Int J Obes (Lond) (2018) 42(4):850–7. doi: 10.1038/ijo.2017.289 PMC5965665 29151596

[17] Li S Yu H Zhang P Tu Y Xiao Y Yang D. The nonlinear relationship between psoas cross-sectional area and bmi: A new observation and its insights into diabetes remission after roux-En-Y gastric bypass. Diabetes Care (2021) 44(12):2783–6. doi: 10.2337/dc20-2907 PMC8669530 34645667

[18] Draznin B Aroda VR Bakris G Benson G Brown FM Freeman R. 2. classification and diagnosis of diabetes: Standards of medical care in diabetes-2022. Diabetes Care (2022) 45(Suppl 1):S17–s38. doi: 10.2337/dc22-S002 34964875

[19] Akaike's Information Criterion. Model selection and model averaging. In: Claeskens G Hjort NL, editors. Cambridge Series in statistical and probabilistic mathematics. Cambridge: Cambridge University Press (2008). p. 22–69.

[20] The Bayesian Information Criterion. Model selection and model averaging. In: Claeskens G Hjort NL, editors. Cambridge Series in statistical and probabilistic mathematics. Cambridge: Cambridge University Press (2008). p. 70–98.

[21] Lawrence JM Divers J Isom S Saydah S Imperatore G Pihoker C. Trends in prevalence of type 1 and type 2 diabetes in children and adolescents in the us, 2001-2017. Jama (2021) 326(8):717–27. doi: 10.1001/jama.2021.11165 PMC8385600 34427600

[22] Barbiellini Amidei C Fayosse A Dumurgier J Machado-Fragua MD Tabak AG van Sloten T. Association between age at diabetes onset and subsequent risk of dementia. Jama (2021) 325(16):1640–9. doi: 10.1001/jama.2021.4001 PMC8080220 33904867

[23] Ellison-Barnes A Johnson S Gudzune K. Trends in obesity prevalence among adults aged 18 through 25 years, 1976-2018. Jama (2021) 326(20):2073–4. doi: 10.1001/jama.2021.16685 PMC8611474 34812876

[24] Twig G Zucker I Afek A Cukierman-Yaffe T Bendor CD Derazne E. Adolescent obesity and early-onset type 2 diabetes. Diabetes Care (2020) 43(7):1487–95. doi: 10.2337/dc19-1988 32321731

[25] Panunzi S De Gaetano A Carnicelli A Mingrone G. Predictors of remission of diabetes mellitus in severely obese individuals undergoing bariatric surgery: Do bmi or procedure choice matter? a meta-analysis. Ann Surg (2015) 261(3):459–67. doi: 10.1097/sla.0000000000000863 25361217

[26] Rubino F Nathan DM Eckel RH Schauer PR Alberti KG Zimmet PZ. Metabolic surgery in the treatment algorithm for type 2 diabetes: A joint statement by international diabetes organizations. Diabetes Care (2016) 39(6):861–77. doi: 10.2337/dc16-0236 27222544

[27] Dale CE Fatemifar G Palmer TM White J Prieto-Merino D Zabaneh D. Causal associations of adiposity and body fat distribution with coronary heart disease, stroke subtypes, and type 2 diabetes mellitus: A mendelian randomization analysis. Circulation (2017) 135(24):2373–88. doi: 10.1161/circulationaha.116.026560 PMC5515354 28500271

[28] Jayedi A Soltani S Motlagh SZ Emadi A Shahinfar H Moosavi H. Anthropometric and adiposity indicators and risk of type 2 diabetes: Systematic review and dose-response meta-analysis of cohort studies. Bmj (2022) 376:e067516. doi: 10.1136/bmj-2021-067516 35042741 PMC8764578

[29] Stefan N Schick F Häring HU. Causes, characteristics, and consequences of metabolically unhealthy normal weight in humans. Cell Metab (2017) 26(2):292–300. doi: 10.1016/j.cmet.2017.07.008 28768170

